# A yeast-based screening assay identifies repurposed drugs that suppress mitochondrial fusion and mtDNA maintenance defects

**DOI:** 10.1242/dmm.036558

**Published:** 2019-02-07

**Authors:** Thomas Delerue, Déborah Tribouillard-Tanvier, Marlène Daloyau, Farnoosh Khosrobakhsh, Laurent Jean Emorine, Gaëlle Friocourt, Pascale Belenguer, Marc Blondel, Laetitia Arnauné-Pelloquin

**Affiliations:** 1 Research Center on Animal Cognition (CRCA) and Center of Developmental Biology (CBD), Center for Integrative Biology (CBI), Toulouse University, CNRS, UPS, 118 route de Narbonne, 31062 Toulouse, France; 2 Institut National de la Santé et de la Recherche Médicale UMR1078, Université de Bretagne Occidentale, Etablissement Français du Sang Bretagne, CHRU Brest, Hôpital Morvan, Laboratoire de Génétique Moléculaire, 29200 Brest, France; 3 Institut de Biochimie et Génétique Cellulaires, CNRS UMR 5095, Université de Bordeaux, 1 rue Camille Saint-Saëns, 33077 Bordeaux, France

**Keywords:** Mitochondrial fusion, Mitochondrial DNA, Hexestrol, Clomifene, Yeast, OPA1

## Abstract

Mitochondria continually move, fuse and divide, and these dynamics are essential for the proper function of the organelles. Indeed, the dynamic balance of fusion and fission of mitochondria determines their morphology and allows their immediate adaptation to energetic needs as well as preserving their integrity. As a consequence, mitochondrial fusion and fission dynamics and the proteins that control these processes, which are conserved from yeast to human, are essential, and their disturbances are associated with severe human disorders, including neurodegenerative diseases. For example, mutations in *OPA1*, which encodes a conserved factor essential for mitochondrial fusion, lead to optic atrophy 1, a neurodegeneration that affects the optic nerve, eventually leading to blindness. Here, by screening a collection of ∼1600 repurposed drugs on a fission yeast model, we identified five compounds able to efficiently prevent the lethality associated with the loss of Msp1p, the fission yeast ortholog of OPA1. One compound, hexestrol, was able to rescue both the mitochondrial fragmentation and mitochondrial DNA (mtDNA) depletion induced by the loss of Msp1p, whereas the second, clomifene, only suppressed the mtDNA defect. Yeast has already been successfully used to identify candidate drugs to treat inherited mitochondrial diseases; this work may therefore provide useful leads for the treatment of optic atrophies such as optic atrophy 1 or Leber hereditary optic neuropathy.

## Abstract (precis)

**Editor's choice:** Mitochondria are conserved among eukaryotes and mutations in the factors that control their dynamics are causal for various human disorders. Hence, yeast models can be used for pharmacological screenings.

## INTRODUCTION

Mitochondrial morphology varies from an interconnected filamentous network to isolated dots, according to cell type and cellular context (Collins and Bootman, 2003). It depends on mitochondrial dynamics, which corresponds to a balance between antagonistic forces of fission and fusion acting on mitochondrial membranes (Bertholet et al., 2016) that was first evidenced in the budding yeast *Saccharomyces cerevisiae* (Sesaki and Jensen, 1999; Bleazard et al., 1999). The mitochondriome thus takes the form of interconnected long filaments when fusion predominates over fission and of isolated dots when fission prevails. Mitochondrial dynamics depends on evolutionarily conserved dynamin-related proteins (DRPs) (Bertholet et al., 2016). Dnm1p/DRP1 (also known as DNM1L) drives mitochondrial outer membrane (OM) fission, whereas Fzo1p/mitofusins and Mgm1p/Msp1p/OPA1 control mitochondrial OM and inner membrane fusion, respectively. Mitochondrial dynamics also underlies the adaptation of the organelle to energetic needs and ensures quality control through the complementation or destruction of damaged mitochondria, while directing cells towards apoptosis in cases of severe defects (Bertholet et al., 2016; Chan, 2012; Labbé et al., 2014). Furthermore, mitochondrial dynamics plays a major role in the maintenance of the mitochondrial DNA (mtDNA) (Vidoni et al., 2013), as evidenced in *S. cerevisiae* in which both mitochondrial morphology defects and mtDNA loss induced by inactivation of mitochondrial fusion could be suppressed by genetically induced loss of mitochondrial fission (Fekkes et al., 2000). The depletion of mtDNA following the inactivation of fusion has also been observed in fission yeast lacking Fzo1p or Mgm1p/Msp1p and in mammalian cells lacking mitofusin 2 (MFN2) or OPA1 (Chen et al., 2007, 2010; Elachouri et al., 2011; Hermann et al., 1998; Jones and Fangman, 1992; Pelloquin et al., 1998; Rapaport et al., 1998; Wong et al., 2000). In addition, DRP1-dependent mitochondrial fission is essential for mtDNA nucleoid structure and distribution (Ban-Ishihara et al., 2013; Ishihara et al., 2015; Murley et al., 2013; Parone et al., 2008).

The inactivation of mitochondrial dynamics is associated with severe diseases, including notably several neurodegenerative disorders (Bertholet et al., 2016). Mutations in the genes encoding MFN2 and OPA1 are responsible for Charcot-Marie-Tooth (CMT) disease and dominant optic atrophy (DOA), respectively (Delettre et al., 2000; Züchner et al., 2004). Mutations in the genes encoding GDAP1 and SLC25A46, two mitochondrial proteins with pro-fission activity, are also linked to CMT disease (Abrams et al., 2015; Baxter et al., 2002). Furthermore, very rare *de novo* mutations of *DRP1* severely impair nervous system development (Fahrner et al., 2016; Sheffer et al., 2016; Waterham et al., 2007), and it was recently shown that some mutations of *DRP1* induce isolated DOA (Gerber et al., 2017). In addition, defects of mitochondrial dynamics are associated with Alzheimer's, Parkinson's and Huntington's diseases (Gao et al., 2017).

Much progress has recently been made towards understanding the molecular mechanisms regulating mitochondrial dynamics, but effective treatment for mitochondrial dynamics-linked diseases is still extremely limited. Recently, a yeast-based assay has been developed for identifying drugs active against human mitochondrial disorders (Couplan et al., 2011). We used the same strategy to search for pharmacological suppressors of mitochondrial fusion defects. Given the link between mitochondrial fusion and mtDNA maintenance, we used the *petite*-negative yeast *Schizosaccharomyces pombe*, which, like mammalian cells and contrary to *S. cerevisiae*, cannot survive without mtDNA (Chen and Clark-Walker, 1999; Schäfer, 2003). Here, we identified, from various repurposed libraries representing ∼1600 drugs, five compounds able to efficiently prevent the lethality associated with the loss of mitochondrial fusion in *S. pombe* consecutive to the inducible loss of Msp1p, the *S. pombe* ortholog of OPA1. We characterized the effects of hexestrol and clomifene, the two most promising drugs, on mitochondrial morphology and mtDNA maintenance in fission yeast. Hexestrol was able to rescue both the mitochondrial fragmentation and mtDNA depletion induced by the loss of Msp1p, whereas clomifene only suppressed the mtDNA defect. We also obtained evidence that the two drugs display two distinct mechanisms of action, as hexestrol, unlike clomifene, does not need the presence of the Msp1p protein for its activity. Furthermore, it appeared that hexestrol might inhibit mitochondrial fission, thereby counterbalancing the effect of Msp1p deficiency on mitochondrial fusion.

## RESULTS

### Identification of molecules preventing the lethality associated with Msp1p inactivation

**Fig. 1.**
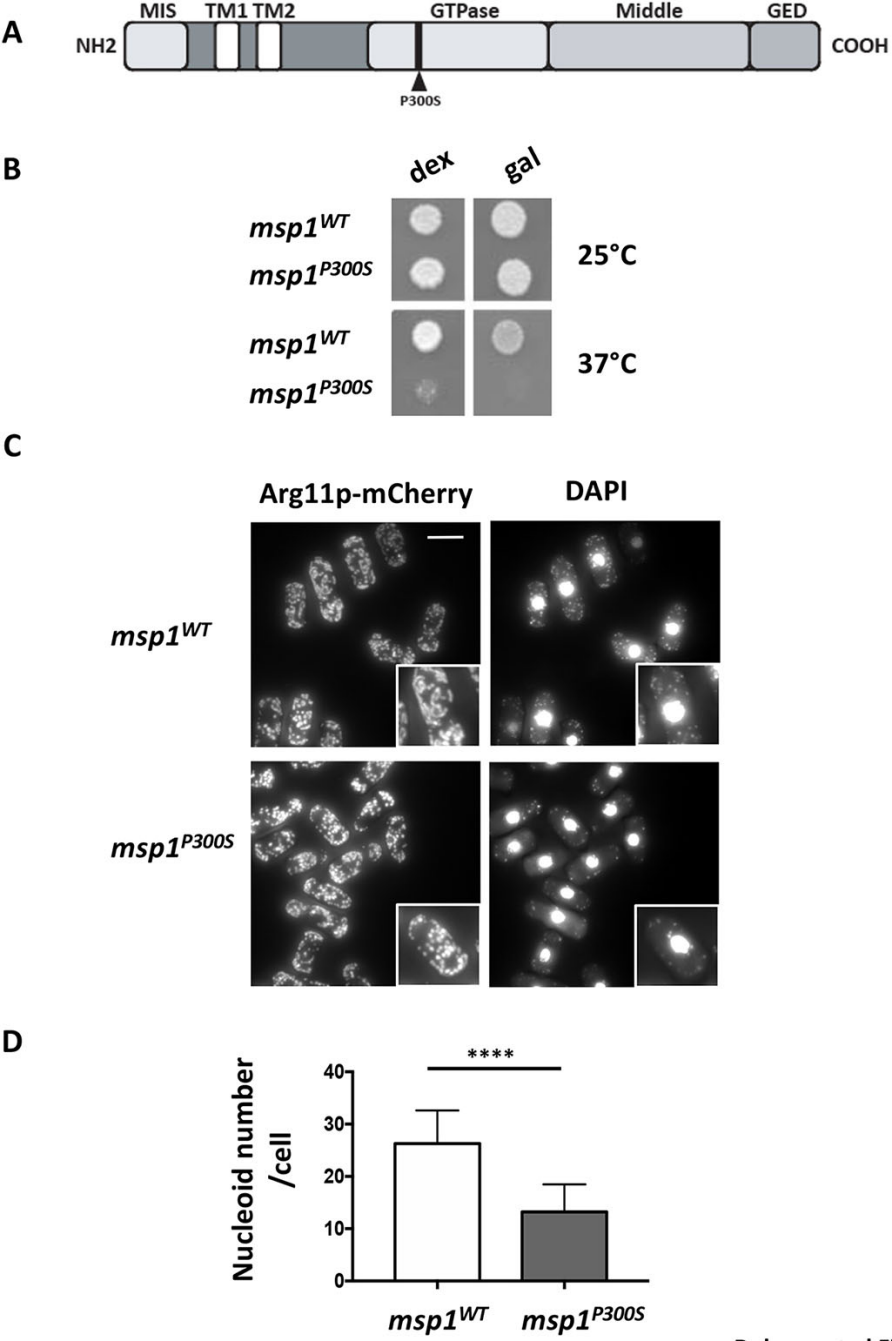
**The P300S thermosensitive mutation in the GTPase domain of Mps1p is lethal for the yeast *S. pombe* grown at restrictive temperature in a galactose-based medium.** (A) Schematic representation of the Msp1p protein and its domains: mitochondrial import sequence (MIS), transmembrane domains (TM1 and TM2), catalytic domain (GTPase), central domain (Middle) and GTPase effector domain (GED). The thermosensitive mutant contains at position 300 a serine residue instead of a proline residue (P300S). (B) Drops, each containing 800 cells of the *msp1^WT^* or *msp1^P300S^* strains, were deposited onto solid agar-based medium containing dextrose (dex) or galactose (gal). The plates were then incubated at 25°C or 37°C for 3 days and photographed. (C) *msp1^WT^* or mutant *msp1^P300S^* strains expressing a version of the mitochondrial protein Arg11p fused to the fluorescent mCherry protein (Arg11p-mCherry) were grown at 37°C for 18 h in galactose liquid medium, fixed and labeled with DAPI before visualization by fluorescence microscopy. Scale bar: 5 µm. High magnifications are shown in insets (×1.7). B and C are representative of five independent experiments. (D) The numbers of mitochondrial nucleoids, visualized as bright dots scattered throughout the cytoplasm in fluorescence microscopy after DAPI staining, were counted in the *msp1^WT^* and *msp1^P300S^* strains cultured at 37°C in galactose liquid medium for 18 h. Data represent the mean±s.d. of three independent experiments, with 50 cells per condition, and were statistically analyzed using a two-tailed unpaired Student's *t*-test (****P<0.0001).

We recently constructed a mutant *S. pombe* strain (*msp1^P300S^*) expressing a thermosensitive version of Msp1p (Delerue et al., 2016). This strain has a point mutation leading to the replacement of the proline residue in position 300 in the Msp1p GTPase domain by a serine residue (Fig. 1A). As expected for a conditional mutation affecting the function of Msp1p, the P300S mutation causes severe growth retardation in dextrose medium at restrictive temperature (Delerue et al., 2016) (Fig. 1B), fragmentation of the mitochondrial network (Delerue et al., 2016), and a decrease in the amount of mtDNA (Delerue et al., 2016). In addition, the *msp1^P300S^* strain was unable to grow at the restrictive temperature in galactose medium, whereas the corresponding strain bearing the wild-type (WT) allele of the *msp1^+^* gene (*msp1^WT^*) grew normally in the same conditions (Fig. 1B). The mitochondrial network of the *msp1^P300S^* strain, as visualized by fluorescence microscopy using the mitochondrial protein Arg11p fused to the fluorescent protein mCherry (Arg11p-mCherry) (Delerue et al., 2016), appeared as dots and more fragmented than that of the *msp1^WT^* strain, which consisted of short filaments (Fig. 1C, left column). Staining with 4′,6-diamidino-2-phenylindole (DAPI) showed that, in the *msp1^P300S^* strain, the number of mitochondrial nucleoids, visualized as bright dots scattered throughout the cytoplasm in fluorescence microscopy, was lower at the restrictive temperature (Fig. 1C, right column). Indeed, the number of nucleoids reached a mean value of ≈13 per cell in the *msp1^P300S^* and was shown to be statistically different from that of the *msp1^WT^*, which reached ≈26 (Fig. 1D)*.*

**Fig. 2.**
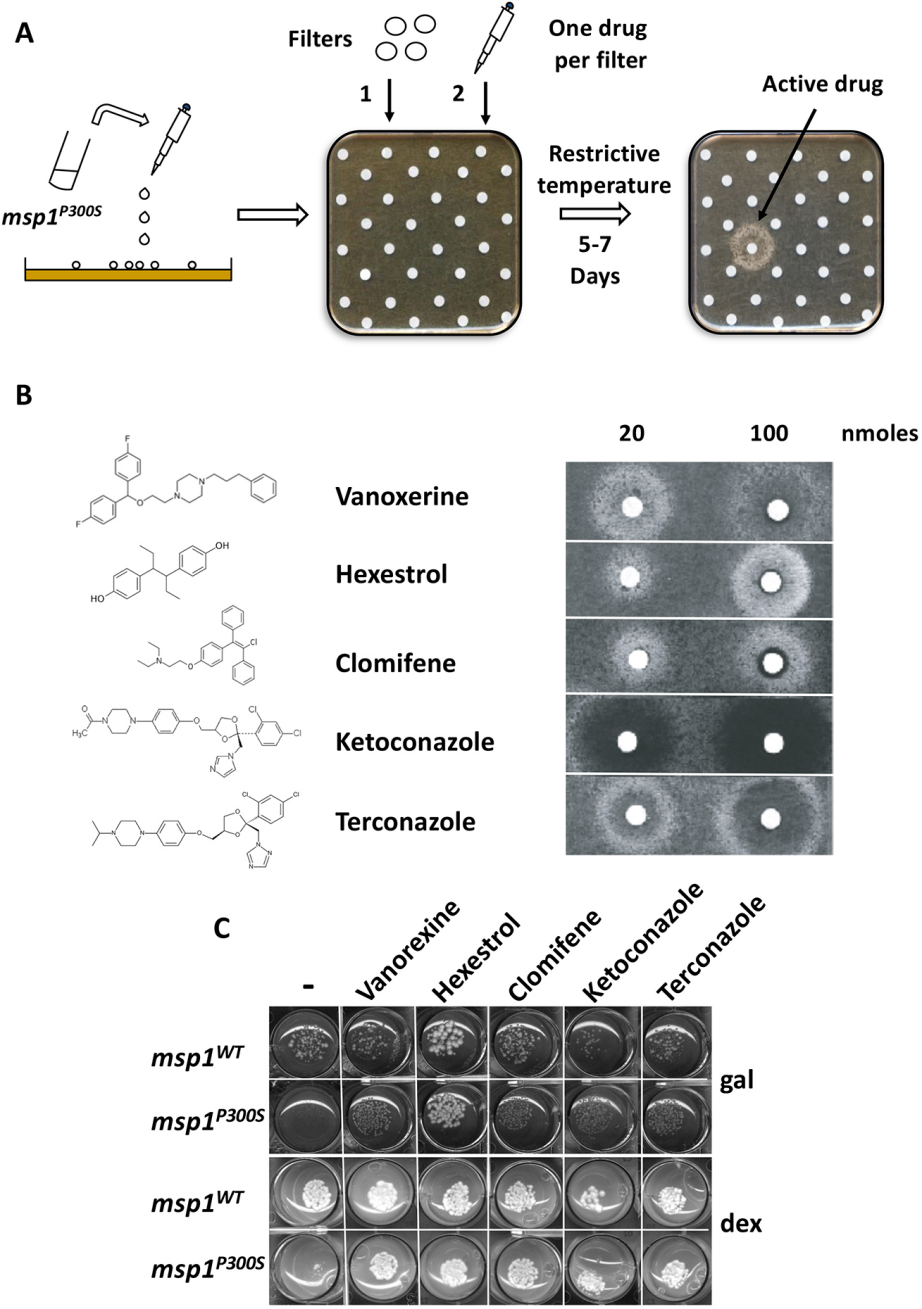
**Drug screening to isolate pharmacological suppressors of lethality of an *msp1^P300S^* strain grown at restrictive temperature in a galactose-based medium.** (A) Experimental strategy: a yeast strain expressing a thermosensitive form of the Msp1p protein (*msp1^P300S^*) was grown at the permissive temperature (25°C) and then spread on agar-based solid medium containing galactose. Then, filters were deposited onto the agar surface and individually loaded with single pharmacological compounds from repurposed drug libraries (3 µl at 10 mM or DMSO as a control onto the top left filter) and Petri plates were then incubated at the restrictive temperature (37°C) for 5-7 days and photographed. (B) The presence of a white halo around the filter indicates yeast growth. The presence of a dark halo indicates the absence of growth and therefore toxicity of the compound at high concentration (close to the filter). The names of the compounds and their chemical structures are indicated. The indicated quantities of drugs were added onto the filters. (C) Drops containing 800 cells expressing WT (*msp1^WT^*) or thermosensitive (*msp1^P300S^*) Msp1p protein were deposited on agar-based solid medium containing either galactose (gal) without (-) or with 6 μM vanoxerine, 30 μM hexestrol, 15 μM clomifene, 1 μM ketoconazole or 9 μM terconazole, or dextrose (dex) without (-) or with 1 μM vanoxerine, 10 μM hexestrol, 15 μM clomifene, 1 μM ketoconazole or 1 μM terconazole, as indicated. The plates were then incubated at 37°C for 3 days and photographed. C is representative of three independent experiments.

We used the lethality of the *msp1^P300S^* mutation in galactose at the restrictive temperature as a readout for a yeast-based pharmacological screening strategy (Couplan et al., 2011) to identify drugs able to suppress the consequences of a defect in Msp1p. Using this simple assay based on a positive readout (restoration of growth), we screened ∼1600 molecules from the Prestwick and TebuBio repurposed drug libraries (Fig. 2A). Briefly, we spread the *msp1^P300S^* strain on solid agar-based galactose medium, and added onto the agar surface filters individually loaded with the various compounds from the tested chemical libraries. We then incubated the plates at the restrictive temperature. Active compounds were identified after 5-7 days of incubation by the halo of yeast growth around the filters where they were deposited. We identified five highly active compounds: vanoxerine, hexestrol, clomifene, ketoconazole and terconazole (Fig. 2B). We then carried out droplet growth tests at the restrictive temperature on galactose and dextrose media, both supplemented with the indicated drugs, to validate their effect. As expected, they abolished the lethality associated with the *msp1^P300S^* mutation in galactose medium (Fig. 2C, top two rows), and also the growth retardation observed in dextrose medium at the restrictive temperature (Fig. 2C, bottom two rows).

### Characterization of the effects of hexestrol and clomifene on mitochondrial morphology and mtDNA maintenance

Msp1p inactivation leads to mitochondrial fragmentation and loss of the mitochondrial genome (Delerue et al., 2016; Guillou et al., 2005; Pelloquin et al., 1998). We therefore investigated whether the drugs identified as able to suppress the growth defect of the *msp1^P300S^* strain also suppress these mitochondria-associated phenotypes. We discarded drugs with antifungal activity (ketoconazole and terconazole) and focused on the two drugs with the lowest toxicity, hexestrol and clomifene, as evidenced by the limited halo of growth inhibition around the filters on which they were deposited compared with the three other drugs (Fig. 2B).

**Fig. 3.**
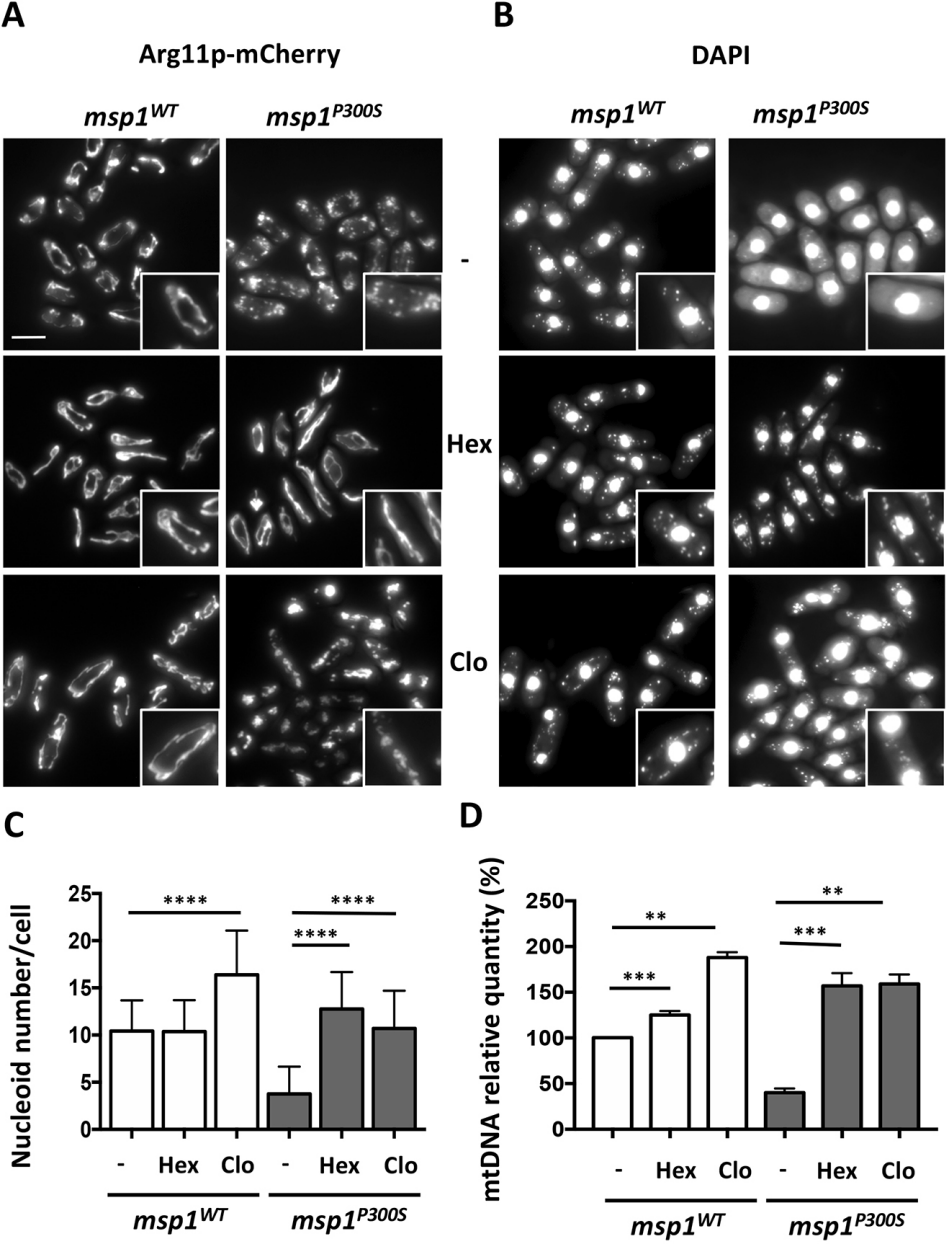
**Effects of hexestrol and clomifene on mitochondrial morphology and maintenance of mtDNA.** (A,B) Yeasts expressing the mitochondrial protein Arg11p fused to the fluorescent mCherry protein (Arg11p-mCherry), together with either WT (strain *msp1^WT^*) or mutated (strain *msp1^P300S^*) Msp1p protein, were cultured at 37°C for 18 h on dextrose liquid medium, without (-) or with 15 μM hexestrol (Hex) or 4 μM clomifene (Clo) as indicated, and then fixed and labeled with DAPI before being observed with a fluorescence microscope. Scale bar: 5 μm. High magnifications are shown in insets (×1.7). A and B are representative of five experiments. (C) The number of mitochondrial nucleoids was counted in the *msp1^WT^* and *msp1^P300S^* strains cultured at 37°C in dextrose liquid medium for 18 h without (-) or with 15 μM hexestrol (Hex) or 4 μM clomifene (Clo), as indicated. Data represent the mean±s.d. of two independent experiments with 50 cells per condition. They were statistically analyzed using Kruskal–Wallis Dunn's multiple comparison tests to compare, for each strain, the values obtained with drugs (Hex, Clo) with that obtained for the control (-) (****P<0.0001). (D) qPCR was performed on DNA extracted from the *msp1^WT^* and *msp1^P300S^* strains cultured at 37°C on dextrose liquid medium for 18 h without (-) or with 15 μM hexestrol (Hex) or 4 μM clomifene (Clo), as indicated. The results of three independent experiments performed in triplicate are expressed as a percentage relative to those obtained for the *msp1^WT^* strain cultured at 37°C (Student's *t*-tests; **P<0.01; ***P<0.001).

**Fig. 4.**
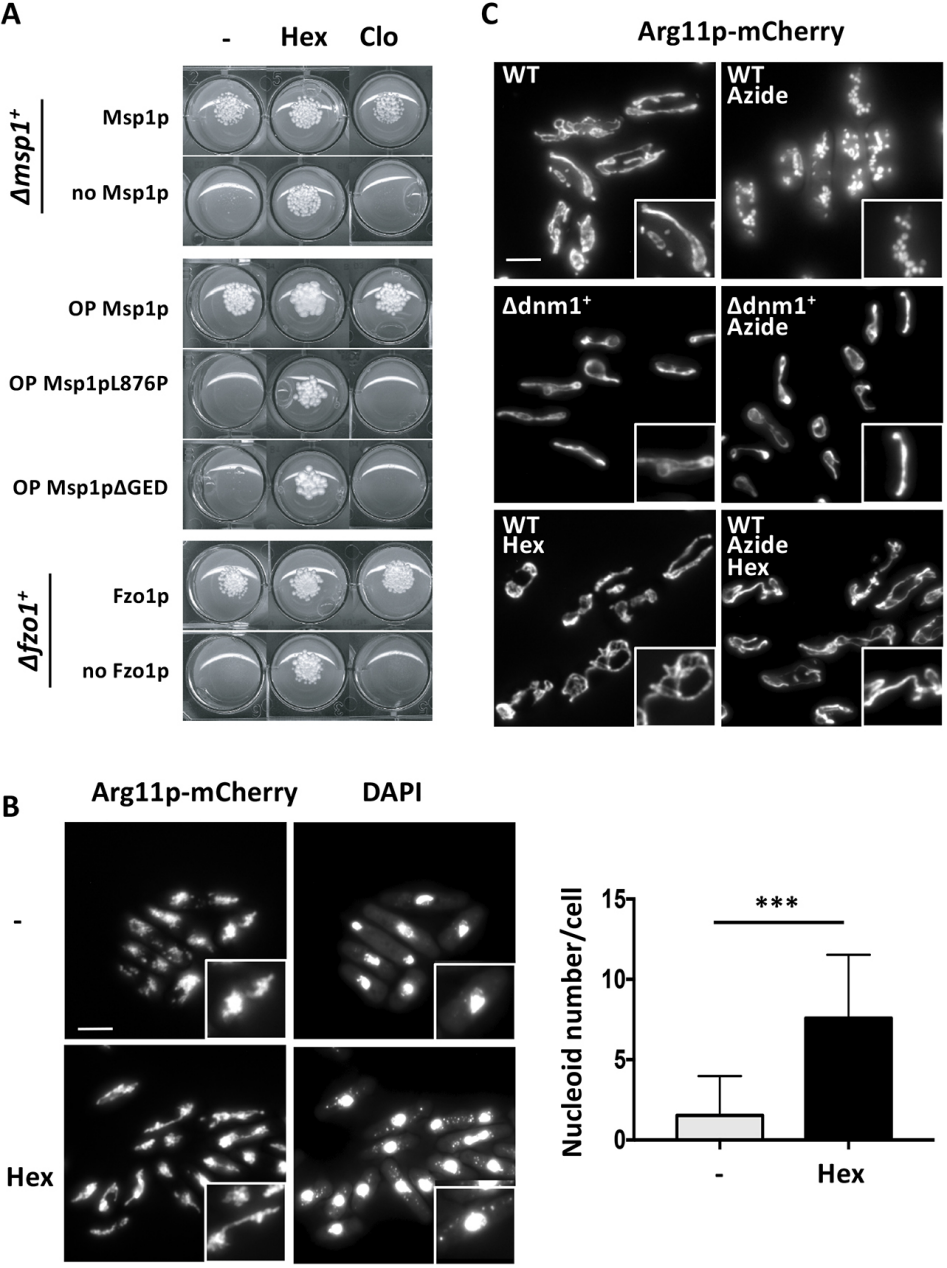
**Mechanisms of action of hexestrol and clomifene.** (A) Yeast strains of the indicated genotypes (Table 1) were cultured on dextrose minimal medium without (-) or with 50 μM hexestrol (Hex) or 15 μM clomifene (Clo) at 25°C for 6 days and then photographed. Top panel: strains with deletions of *msp1^+^* (Δ*msp1^+^*) ectopically expressing *msp1^+^*, or not, under the control of the *nmt1* promoter. Msp1p is produced in the absence of thiamine (Msp1p), but not in its presence (no Msp1p). Middle panel: WT strains ectopically overexpressing a WT form of *msp1^+^*, or a form containing a mutated GED domain, under the control of the *nmt1^+^* promoter. WT Msp1p (OP Msp1p), Msp1p with the L876P mutation (OP Msp1pL876P) or Msp1p with a deletion of the last 50 amino acids of the protein (OP Msp1pΔGED) were overexpressed in the absence of thiamine. Bottom panel: strains with deletions of *fzo1^+^* (Δ*fzo1^+^*) ectopically expressing *fzo1^+^* under the control of the *nmt1*^+^ promoter. Fzo1p (Fzo1p) is produced in the absence of thiamine, whereas it is not expressed (no Fzo1p) in its presence. (B) Yeasts with deletion of *msp1^+^* (Δ*msp1^+^*) expressing the mitochondrial protein Arg11p fused to the fluorescent mCherry protein (Arg11p-mCherry), for which the ectopic expression of *msp1^+^* was abolished by addition of thiamine (no Msp1p), were cultured at 25°C in dextrose minimal liquid medium for 72 h with or without 50 μM hexestrol (Hex), and stained with DAPI before observation under a fluorescence microscope. Left column: representative pictures of Arg11p-mCherry and DAPI staining. Scale bar: 5 μm. High magnifications are shown in insets (×1.7). Right column: the number of mitochondrial nucleoids was counted in yeasts with deletion of *msp1^+^* (Δ*msp1^+^*) cultured at 25°C in dextrose liquid medium for 72 h with thiamine (no Mps1p) and without (-) or with 50 μM hexestrol (Hex). Data represent the mean±s.d. of three independent experiments, with 60 cells per condition, and were statistically analyzed by a two-tailed unpaired Mann–Whitney test (***P<0.001). (C) A WT strain and a strain deleted for the *dnm1^+^* gene (Δ*dnm1^+^*), expressing the mitochondrial protein Arg11p fused to the fluorescent mCherry protein (Arg11p-mCherry) were cultured in dextrose liquid medium at 25°C with or without 15 μM hexestrol (Hex) for 18 h as indicated. Sodium azide (Azide) was then added to a final concentration of 0.02% w/v, the cultures were incubated for 30 min, and the cells were fixed and observed by fluorescence microscopy. Scale bar: 5 μm. High magnifications are shown in insets (×1.7). A, B and C are representative of three experiments.

The *msp1^WT^* strain had a filamentous mitochondrial network in dextrose at the restrictive temperature (Delerue et al., 2016) (Fig. 3A). By contrast, the mitochondria of the *msp1^P300S^* strain were fragmented. Strikingly, the mitochondrial network in the *msp1^P300S^* strain was no longer fragmented in the presence of hexestrol, whereas the mitochondria remained fragmented and tended to cluster in the presence of clomifene. In addition, hexestrol induced mitochondrial hyperfilamentation in the *msp1^WT^* strain cultured at 37°C, whereas clomifene did not (Fig. 3A), and this effect was more pronounced at the permissive temperature (Fig. 4C).

**Table 1.**
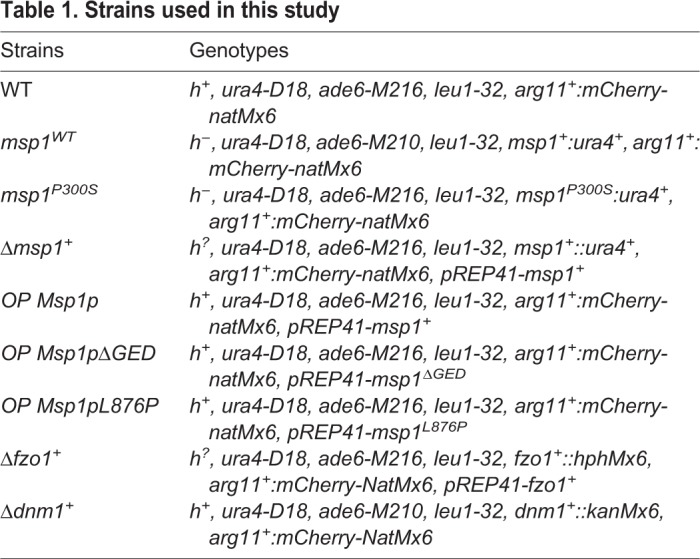
**Strains used in this study**

In the absence of drug, the mitochondrial nucleoids were clearly detected in the *msp1^WT^* strain cultured in dextrose medium at the restrictive temperature, whereas they were barely visible, if at all, in the *msp1^P300S^* strain (Delerue et al., 2016) (Fig. 3B). Strikingly, the *msp1^P300S^* strain cultured at a restrictive temperature, but in the presence of hexestrol or clomifene, contained normal numbers of mitochondrial nucleoids (Fig. 3C). In the *msp1^WT^* strain, clomiphene induced an increase in the nucleoid number, whereas hexestrol had no effect (Fig. 3C).

We then measured the amount of mtDNA relative to nuclear DNA by quantitative PCR (qPCR) (Fig. 3D). The results are expressed relative to those for the *msp1^WT^* strain grown at the restrictive temperature without drugs. Hexestrol and clomifene increased the amount of mtDNA present in the *msp1^WT^* strain at the restrictive temperature and restored mtDNA levels in the *msp1^P300S^* strain, to levels greater than those in the untreated *msp1^WT^* strain.

Of note, similar effects of hexestrol and clomifene on the mitochondrial morphology and mtDNA were observed for the *msp1^P300S^* strain in galactose medium at 37°C (Fig. S1).

Altogether, these results indicate that both hexestrol and clomifene were able to suppress the loss of mitochondrial DNA due to defect in Msp1p, whereas only hexestrol was also able to suppress the mitochondria fragmented morphological phenotype. Hence, these results also suggest that hexestrol, which led by itself to mitochondrial hyperfilamentation in WT cells, promoted mitochondrial fusion, whereas clomifene did not. Importantly, such an effect of hexestrol could result from either activation of fusion or inhibition of fission.

### Characterization of the mechanisms of action of hexestrol and clomifene

We characterized the mode of action of hexestrol and clomifene by first determining whether the presence of Msp1p was essential for the activity of these drugs. We used a strain with a deletion of the *msp1^+^* gene (*Δmsp1^+^*), thus expressing Msp1p ectopically under the control of the *nmt1^+^* inducible promoter (Guillou et al., 2005). As a consequence, *msp1^+^* is expressed in the absence of thiamine (Msp1p) and repressed in its presence (no Msp1p). As expected (Guillou et al., 2005; Pelloquin et al., 1998), the *Δmsp1^+^* strain did not grow in dextrose medium in the presence of thiamine (i.e. in the absence of Msp1p; no Msp1p in Fig. 4A, top panel). Strikingly, hexestrol abolished the lethality due to the total loss of *msp1^+^* gene expression, whereas clomifene did not (Fig. 4A, top panel). The effects of hexestrol on mitochondrial morphology and the maintenance of mtDNA in the *Δmsp1^+^* strain were then analyzed (Fig. 4B). As expected (Guillou et al., 2005), the mitochondria were fragmented and clustered and almost all of the cells were lacking nucleoids (mean of ≈0.8 nucleoids per cell, Fig. 4B, left column) in dextrose medium in the presence of thiamine (i.e. in the absence of Msp1p). In these conditions, but in the presence of hexestrol, the mitochondriome consisted mostly of long and aggregated filaments, and nucleoids were clearly visible (mean of ≈8 nucleoids per cell, Fig. 4B, right column).

We then investigated whether hexestrol and clomifene abolish the lethality induced by mutations of the Msp1p GTPase effector domain (GED), which, as the GTPase domain, was shown to be essential. We indeed previously showed that expression of Msp1p mutants bearing mutation in the GTPase domain or in the GED domain were unable to complement the deletion of the *msp1^+^* gene (Guillou et al., 2005). Furthermore, we also previously showed that overexpression of Msp1p bearing a GED deletion had a dominant-negative effect (Guillou et al., 2005). We thus overexpressed either a deletion mutant (ΔGED) (Guillou et al., 2005) or a point mutant (L876P) of the GED domain in a strain carrying the WT *msp1^+^* gene. As expected, overexpression in *S. pombe* of any of these mutated forms of Msp1p was lethal, whereas overexpression of WT Msp1p was not (Fig. 4A, middle panel). Strikingly, hexestrol abolished the lethality due to the overexpression of GED-domain mutants, whereas clomifene did not (Fig. 4A, middle panel).

Finally, we also investigated whether hexestrol and clomifene abolished the lethality induced by the inactivation of the second fusion actor, Fzo1p. A strain in which deletion of the *fzo1^+^* gene (*Δfzo1^+^*) was complemented by ectopic expression of Fzo1p under the control of the *nmt1^+^* promoter did grow when Fzo1p was induced (Fzo1p in Fig. 4A, bottom panel), but not when it was repressed by addition of thiamine (no Fzo1p in Fig. 4A, bottom panel). The *fzo1^+^* gene is, therefore, essential in *S. pombe*, as already reported for its budding yeast counterpart (Hermann et al., 1998; Rapaport et al., 1998). Again, hexestrol abolished the lethality associated with the loss of Fzo1p, whereas clomifene did not (Fig. 4A, bottom panel).

Hexestrol does not, therefore, require the Msp1p and Fzo1p fusogenic proteins to function. Hence, similarly to the inactivation of fission, hexestrol can abolish mitochondrial fusion defects. For this reason, we investigated whether hexestrol inhibited mitochondrial fission, using sodium azide, an inhibitor of complex IV of the respiratory chain that induces the fission of mitochondria. The mitochondrial network of a WT strain was already fragmented after 15 min of treatment by sodium azide (Fig. 4C, top row). This fragmentation was dependent on the fission protein Dnm1p, because it did not occur in a strain lacking the *dnm1^+^* gene (*Δdnm1^+^*) treated with sodium azide (Fig. 4C, middle row). In the WT strain untreated with sodium azide, hexestrol induced mitochondrial hyperfilamentation (Fig. 4C, bottom left image) and restored a filamentous network in the presence of sodium azide, which normally induced fragmentation (Fig. 4C, bottom right image). Together, these results indicate that hexestrol is an inhibitor of mitochondrial fission, hence impeding the mitochondrial fragmentation observed in strains defective for fusion because of inactivation of either Msp1p or Fzo1p.

## DISCUSSION

We identified pharmacological compounds that abolished phenotypes associated with inactivation of Msp1p, the *S. pombe* ortholog of OPA1 a GTPase involved in DOA. In doing so, we screened chemical libraries of repurposed drugs with a yeast strain expressing, as a sole source of Msp1p, a thermosensitive version of Msp1p protein containing a point mutation affecting its GTPase domain (*msp1^P300S^*). At the permissive temperature, this strain behaved like those bearing a WT *msp1^+^* allele, whereas, at the restrictive temperature, it displayed a fragmented mitochondrial network and a significant decrease in mtDNA. In addition, the *msp1^P300S^* strain displayed a growth delay in dextrose medium and lethality in galactose medium at the restrictive temperature. The loss of viability of the *msp1^P300S^* strain in galactose medium allowed us to unambiguously screen two chemical libraries, regrouping ∼1600 repurposed compounds that represent most US Food and Drug Administration-approved drugs, and to identify five drugs able to efficiently abolish this phenotype: vanoxerine, hexestrol, clomifene, ketoconazole and terconazole. Hexestrol and clomifene are two non-steroidal estrogens and vanoxerine is a dopamine transporter antagonist that blocks cardiac potassium and sodium ion channels. Ketoconazole and terconazole are antifungal drugs of the imidazole family that act by inhibiting ergosterol (the yeast equivalent of cholesterol) synthesis.

We characterized, in some detail, the effects of two of these five drugs, hexestrol and clomifene, as, in addition to efficiently suppressing the growth defect of the *msp1^P300S^* strain, they present less toxicity at high concentrations. Hexestrol has been used for years to treat estrogen deficiency and is one of the most potent known estrogens (Chamkasem and Toniti, 2015; Solmssen, 1945). Clomifene induces ovulation and has been used as such to treat various cases of female infertility (Kistner, 1965; Wilkes and Murdoch, 2012). Using our screening assay, we showed that tamoxifen and other molecules with estrogenic activity, which are, or not, structurally related to tamoxifen, are not able to suppress the lethality of the *msp1^P300S^* strain in galactose medium (Table S1), suggesting that the effect of clomifene and hexestrol is not related to their estrogenic properties.

Hexestrol and clomifene both suppressed defects in nucleoids and mtDNA amounts in the *msp1^P300S^* strain grown at the restrictive temperature. They also increased the amount of mtDNA in WT yeasts. Such an effect on mtDNA levels, in petite-negative cells, probably explains the restoration of viability and growth rate of the *msp1^P300S^* strain cultured at restrictive temperature. Of note, the fusogenic and mtDNA maintenance functions of Msp1p can hardly be separated and, as a consequence, the determination of the essential or non-essential nature of the fusogenic function of Msp1p is a puzzling question. In a previous work (Diot et al., 2009), we showed that overexpression of Msp1p lacking its first transmembrane domain leads to mitochondrial fragmentation but not to mtDNA loss, while overexpression of a form of Msp1p lacking its second transmembrane domain leads to mitochondrial fragmentation, loss of mtDNA and cell death. Here, we showed that, in the presence of clomifene, the viability of the *msp1^P300S^* strain was restored, whereas its mitochondrial network remained fragmented. Altogether, these two studies thus indicate that the fusogenic function of Msp1p is not essential for *S. pombe* survival, at least in basal conditions. In sharp contrast, hexestrol also abolished the fragmentation of the mitochondrial network. This difference suggests that the modes of action of these two compounds are different. Hexestrol and clomifene may, therefore, represent ideal tools for studying separately the two functions of Msp1p.

Interestingly, high-throughput chemogenomic studies have shown that hexestrol enhances the growth of diploid budding yeast strains harboring heterozygous mutation of *mgm1^+^*, the *S. cerevisiae* homolog of the *msp1^+^* gene (Hillenmeyer et al., 2008). In line, here we found that hexestrol abolished the lethality, mitochondrial fragmentation and mtDNA loss caused by a total loss of Msp1p. This drug thus did not act directly on Msp1p. Accordingly, hexestrol also abolished the lethality associated with a total loss of Fzo1p. This suggests that hexestrol may act on a mechanism counteracting the effects of the inactivation of mitochondrial fusion, similarly to the inactivation of fission. Consistent with this hypothesis, we found that hexestrol abolished Dnm1p-dependent fragmentation of the mitochondrial network induced by sodium azide and promoted hyperfilamentation of mitochondria when used alone. This suggests that Dnm1p-dependent mitochondrial fission is the target of hexestrol. If this effect of hexestrol is conserved in mammals, this drug could provide new avenues for the treatment of various mitochondrial disorders, as proposed for mdivi-1, a fission inhibitor directly targeting DRP1 (Cassidy-Stone et al., 2008; Lackner and Nunnari, 2010).

Unlike hexestrol, clomifene did not abolish the lethality associated either with a total loss of Msp1p, or induced by the overexpression of dominant-negative mutants of Msp1p, or by the total loss of Fzo1p. Therefore, clomifene may act directly on Msp1p, and, as such, most probably requires a minimal residual Msp1p activity to exert its suppressive activity. Of note, the P300S mutation is not located in the GTP-binding site of the GTPase domain and, as a consequence, the Msp1p^P300S^ protein may still possess some GTPase activity that can allow clomifene to act.

Chemogenomic studies have led to the identification of several molecules, including haloperidol, with modes of action potentially similar to that of clomifene, i.e. the ability to inhibit Erg2p, a key enzyme in ergosterol biosynthesis in yeast (Parsons et al., 2006). Hence, we tested haloperidol in our various Msp1-based assays and found it able to abolish the growth retardation of the *msp1^P300S^* strain and the loss of mtDNA in dextrose medium at the restrictive temperature, but unable to prevent fragmentation of the mitochondrial network (Fig. S2). Clomifene and haloperidol may, therefore, have modes of action – interfering with the ergosterol pathway – similar to ketoconazole and terconazole, two other highly active drugs that we identified in our initial screening (Fig. 2). Reinforcing this hypothesis, we found that no less than eight additional drugs that target ergosterol biosynthesis, and that correspond to all other imidazole antifungal drugs from the ∼1600 repurposed drugs screened, were also able to rescue, to various extents, the lethality induced by the inactivation of Msp1 in galactose medium (Fig. S3). Furthermore, naftidine, which does not belongs to the imidazole family but also targets ergosterol biosynthesis, was active as well (Fig. S3). Finally, clomifene was shown to decrease the content of sterols *in S. cerevisiae* (Řezanka et al., 1985). Like cholesterol in mammals, this sterol is important in yeast and controls membrane fluidity and permeability (Iwaki et al., 2008; Parks et al., 1995). Ergosterol is essential for mitochondria, despite its low abundance in the membranes of these organelles. Indeed, mitochondrial morphology is altered in the absence of enzymes of the ergosterol biosynthetic pathway (Altmann and Westermann, 2005). The importance of ergosterol in the specific targeting of proteins anchored to the mitochondrial OM was recently highlighted (Krumpe et al., 2012). Hence, by acting on ergosterol metabolism, clomifene may modify the organization of mitochondrial membranes and/or the localization of membrane mitochondrial proteins, which in turn may affect the anchoring of nucleoids to the internal mitochondrial membrane, and, thereby, their stability or replication (Chen and Butow, 2005; Hayward et al., 2013).

Our approach, which involves screening drug candidates in yeast models of mitochondrial diseases, has already proved efficient to isolate compounds active in patient-derived cells, indicating that yeast may be used successfully to identify candidate drugs to treat inherited mitochondrial diseases (Couplan et al., 2011; Lasserre et al., 2015). Hence, hexestrol and clomifene may represent candidate drugs for the treatment of DOA caused by mutations of *OPA1*, the mammalian homolog of Msp1p. In this context, it may be informative to assess the ability of hexestrol and clomifene to abolish the various defects induced by the loss of OPA1 in primary cortical neurons (Bertholet et al., 2013), in skin fibroblasts from DOA patients (Olichon et al., 2007) and in murine models carrying mutations of the *Opa1* gene (Alavi et al., 2007). Hexestrol and clomifene are particularly interesting because, as drugs already in use in humans for the treatment of estrogen deficiency, data concerning their bioavailability and toxicity are available. Therefore, their repositioning for the treatment of DOA, or other mitochondrial-linked optic neuropathies such as Leber hereditary optic neuropathy, may be envisioned.

## MATERIALS AND METHODS

### Yeast strains and cultures

The *S. pombe* yeast strains used in this study are listed in Table 1. The *msp1^WT^* and *msp1^P300S^* strains were grown at 25°C or 37°C in rich medium containing 1% yeast extract, 2% peptone and 0.1% dextrose supplemented with either 3% dextrose or 3% galactose. *msp1^+^*- or *fzo1^+^*-deleted strains and WT strains overexpressing WT or mutated Msp1p were grown at 25°C in minimal medium (EMM; Bio101, La Jolla, CA, USA) containing dextrose (2%) and supplemented with 225 µg/l adenine, leucine or uracil and 4 µM thiamine, when required. Hexestrol (C_18_H_22_O_2_, Sigma-Aldrich) and clomifene citrate (C_26_H_28_ClNO.C_6_H_8_O_7_, Sigma-Aldrich), as well as vanoxerine (C_28_H_32_F_2_N_2_O·2HCl, Sigma-Aldrich), ketoconazole (C_26_H_28_Cl_2_N_4_O_4_, Sigma-Aldrich), terconazole (C_26_H_31_Cl_2_N_5_O_3_, Sigma-Aldrich) were diluted in dimethyl sulfoxide (DMSO) and added at the concentrations indicated in the figure legends. Effective concentrations were determined by dose responses experiments for each condition, i.e. culture in solid or liquid conditions, in rich medium containing dextrose or galactose, or in minimal medium, at 25°C or 37°C. The same quantity of DMSO was added to the controls, indicated in the figures as ‘(-)’ (without drug).

### Cytological observations

For mitochondrial morphology observations, *S. pombe* cells producing the mitochondrial protein Arg11p tagged with mCherry (Delerue et al., 2016) were fixed in 3.7% formaldehyde for 10 min. For DAPI staining, cells were fixed by incubation in 3.7% formaldehyde for 10 min and were then incubated with 3 µg/ml DAPI and 30% ethanol for 10 min. Cells were observed under a Nikon Eclipse 80*i* microscope (100× objective) and images were taken with the software NIS element AR3.2 (https://www.microscope.healthcare.nikon.com/).

### qPCR

Total cellular DNA was extracted from *S. pombe* spheroplasts (Chu et al., 2007) and amplified by real-time qPCR using Bio-Rad reagents and apparatus (CFX C1000 thermal cycler, CFX96TM real-time system). The ratio of mtDNA to nuclear DNA was determined using previously described primers (Delerue et al., 2016). Individual DNA samples were analyzed on 96-well plates in parallel with calibration curves (five dilutions) for the determination of mitochondrial and nuclear primer pair efficiencies. All experiments were performed in triplicate.

### Drug screening

We screened 1120 molecules from the Prestwick chemical library (http://www.prestwickchemical.com/libraries-screening-lib-pcl.html) and 640 molecules from the TebuBio chemical library, all these compounds being drugs already on the market, for the restoration of yeast *msp1^P300S^* strain viability in galactose medium at 37°C, according to the protocol shown in Fig. 2A and already described for *S. cerevisiae* (Bach et al., 2003, 2006; Couplan et al., 2011).

### Statistical analysis

Data were statistically treated using GraphPad Prism software (graphpad.com). Student's *t*-test (Fig. 1), Kruskal–Wallis test (Fig. 3) and Mann–Whitney test (Fig. 4) were used to compare the numbers of nucleoids per cell. Student's *t*-tests were used to compare the quantities of mtDNA per cell (Fig. 3). *P<0.05; **P<0.01; ***P<0.001; ****P<0.0001.

## Supplementary Material

Supplementary information
