# Supplementary material for: A yeast-based screening assay identifies repurposed drugs that suppress mitochondrial fusion and mtDNA maintenance defects
Source: Dis Model Mech. 2019 Feb 7;12(2):dmm036558. doi: 10.1242/dmm.036558 (PMC6398489; doi:10.1242/dmm.036558)
Supplement: Supplementary information [file dmm-12-036558-s1.pdf]

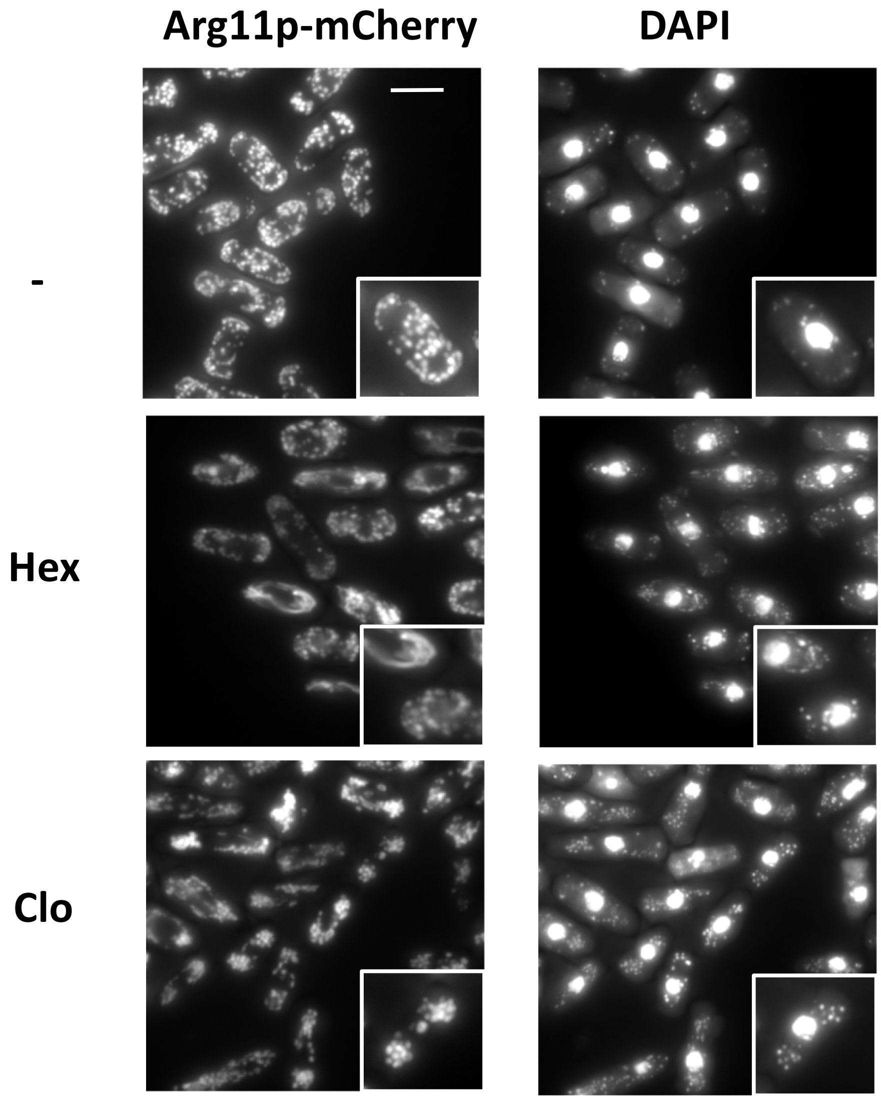

Fig. S1. Effect of hexestrol and clomifene on mitochondrial morphology and mtDNA in galactose medium

Yeast cells expressing the mitochondrial protein Arg11p fused to the fluorescent mCherry protein (Arg11p-mCherry), together with mutated Msp1p (strain *msp1*^{P300S}) were cultured at 37°C in galactose medium for 18 hours without (-) or with 20 µM hexestrol (Hex) or 25 µM clomifene (Clo) as indicated and observed by fluorescence microscopy after fixation and staining with DAPI. Bar represents 5 µm. High magnifications are shown in insert (x1.7). This Figure is representative of 3 experiments.

Disease Models & Mechanisms • Supplementary information

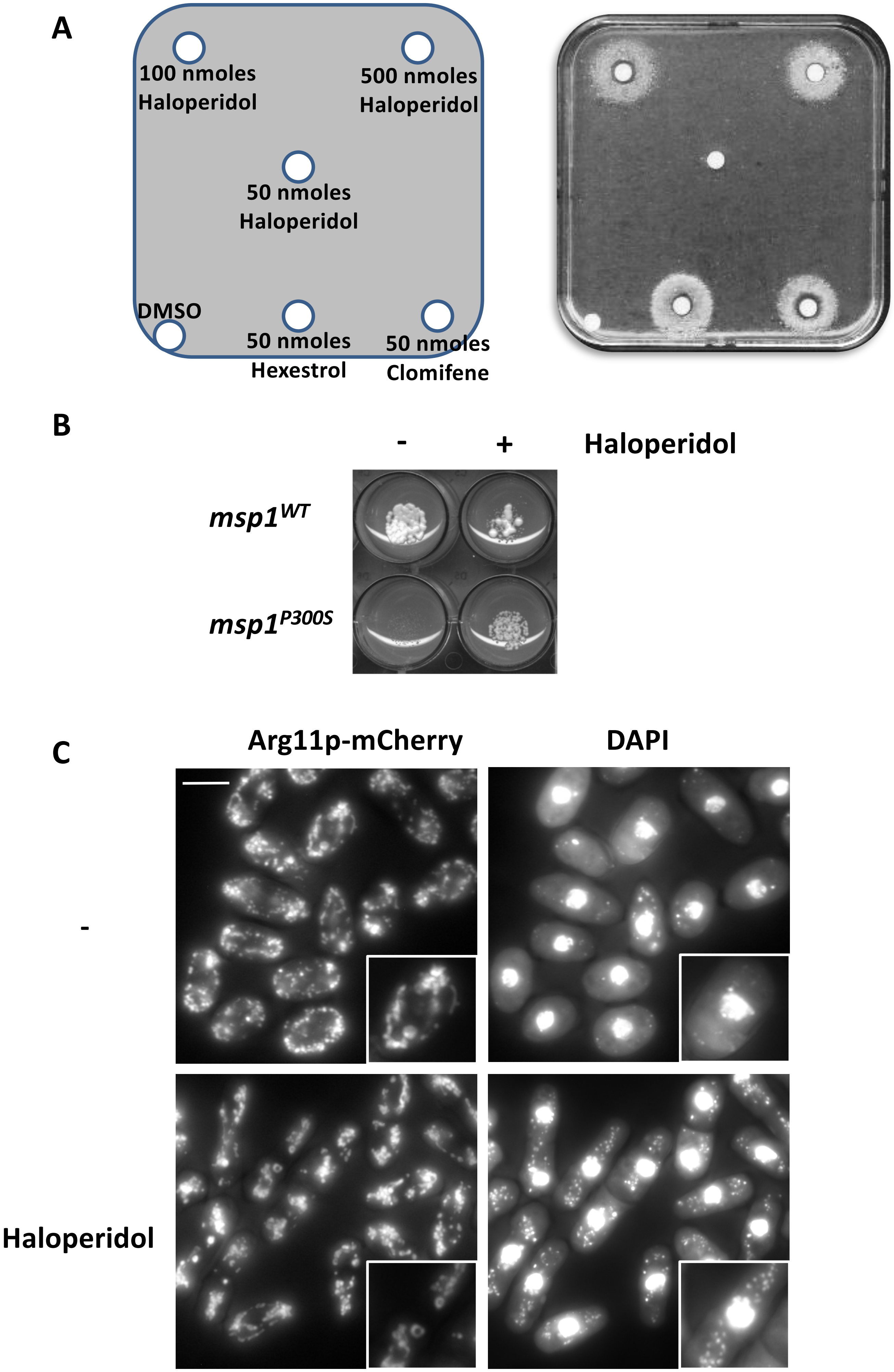
A
100 nmoles Haloperidol
500 nmoles Haloperidol
50 nmoles Haloperidol
DMSO
50 nmoles Hexestrol
50 nmoles Clomifene
B
- + Haloperidol
msp1^WT
msp1^P300S
C
Arg11p-mCherry
DAPI
-
Haloperidol

Fig. S2. Effect of haloperidol on growth, mitochondrial morphology, and mtDNA maintenance of *msp1*^{P300S} mutant strain

A. *msp1*^{P300S} grown at the permissive temperature (25°C) was then spread on agar-based solid medium containing galactose. Then, filters individually loaded with the indicated quantities ofof hexestrol, clomifene or haloperidol dissolved in DMSO or with DMSO alone as a negative control were deposited onto the agar surface and Petri plates were then incubated at the restrictive temperature (37°C) for seven days and photographed.

B. Drops containing 800 cells expressing wild type (strain *msp1*^{WT}) or thermosensitive (strain *msp1*^{P300S}) Msp1p, were deposited on solid agar-based medium containing dextrose, without (-) or with (+) 15 µM haloperidol as indicated and incubated for three days at 37°C and photographed.

C. Yeasts expressing the mitochondrial protein Arg11p fused to the fluorescent mCherry protein (Arg11p-mCherry), together with mutated Msp1p (strain *msp1*^{P300S}), were cultured at 37°C for 18 hours in dextrose liquid medium, without (-) or with 15 µM haloperidol as indicated, fixed and stained with DAPI and then visualized by fluorescence microscopy. Bar represents 5 µm. High magnifications are shown in insert (x1.7).

B and C are representative of 3 experiments.

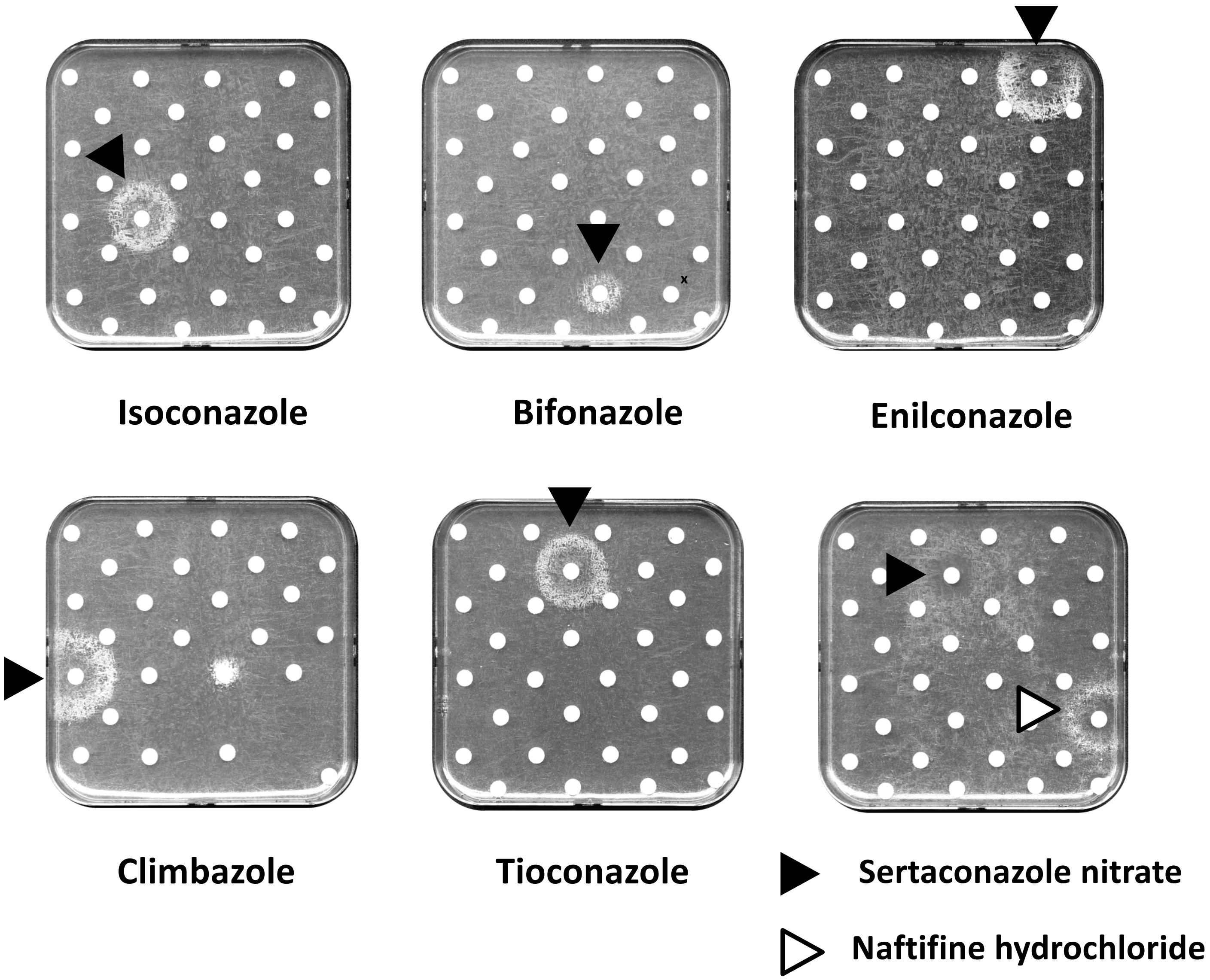

Disease Models & Mechanisms • Supplementary information

Fig. S3. Imidazole antifungals targeting ergosterol biosynthesis are active in the primary screen based on *msp1*^{P300S} thermosensitive strain

Photography of a selection of Petri plates from the primary screen based on the *msp1*^{P300S} strain are shown. Briefly, a yeast strain expressing a thermosensitive form of the Msp1p protein (*msp1*^{P300S}) was grown at the permissive temperature (25°C) and then spread on agar-based solid medium containing galactose. Then, filters were deposited onto the agar surface and individually loaded with single pharmacological compounds from repurposed drug libraries (3 µl at 10 mM or DMSO as a control onto the top left filter) and Petri plates were then incubated at the restrictive temperature (37°C) for five to seven days and photographed. The presence of a white halo around the filter indicates yeast growth and therefore suppression of the growth defect induced by the *msp1*^{P300S} mutation. The presence of a dark halo indicates inhibition of residual growth and thus toxicity of the compound at high concentration (close to the filter). The names of the antifungal compounds are given and the filters where they were deposited indicated by a black arrow head. All the imidazole antifungals from the two chemical libraries of repurposed drugs screened (Prestwick and TebuBio) are active in this primary screen. In addition, Naftifine hydrochloride (white arrowhead), which does not belong to the imidazole family but also targets ergosterol biosynthesis, is active as well (lower plate).

Disease Models & Mechanisms • Supplementary information

Table S1.

Drugs	Rescue the lethality in *msp1*^{P300S} strain
Clomifene	*yes*
Hexestrol	*yes*
Tamoxifen	*no*
4-hydroxytamoxifene	*no*
Torimifene	*no*
Raloxifene	*no*
Fulvestrant	*no*
Estradiol	*no*

Disease Models & Mechanisms • Supplementary information
